# Deep Intramuscular Vascular Malformations in Pediatric Klippel–Trenaunay Syndrome Mimicking Maffucci Syndrome: A Case Report

**DOI:** 10.1002/ccr3.73825

**Published:** 2026-10-29

**Authors:** Syeda Simrah Shah, Barka Sajid, Muzainah Tabassum, Mirha Ali, Taher Janoowala, Tehreem Farooq, Muhammad Ahsan, Mirza Mohammad Ali Baig, Mirza Daniyal Baig, Mohammed Hammad Jaber Amin

**Affiliations:** ^1^ Dow Medical College Karachi Pakistan; ^2^ Jinnah Sindh Medical University Karachi Pakistan; ^3^ Shaheed Mohtarma Benazir Bhutto Medical College Karachi Pakistan; ^4^ Islamic International Medical College Riphah International University Rawalpindi Pakistan; ^5^ Faculty of Medicine Alzaiem Alazhari University Khartoum Sudan

**Keywords:** case report, intramuscular vascular malformation, Klippel–Trenaunay syndrome, Maffucci syndrome, multimodal imaging

## Abstract

Klippel‐Trenaunay syndrome (KTS) is a rare congenital vascular overgrowth disorder characterized by capillary and venous or lymphatic malformations with limb hypertrophy. Deep intramuscular involvement in children is uncommon and may resemble other vascular‐overgrowth syndromes. We report a 12‐year‐old boy with right lower‐limb swelling since birth, recurrent cellulitis, chronic ulceration and purulent discharge, progressive limb enlargement, and four previous soft‐tissue excisions. Examination showed bilateral nodular ulcerated lesions and vascular skin changes. Laboratory testing demonstrated persistent microcytic anemia and raised inflammatory markers. Doppler ultrasonography identified soft‐tissue hypertrophy, macrodactyly, calcified cavernous vascular lesions, superficial venous dilatation, incompetent perforators, and venous reflux, while deep veins remained patent. Contrast‐enhanced magnetic resonance imaging showed extensive intramuscular malformations involving the gastrocnemius and tibialis muscles without marrow or joint involvement. The calcified lesions initially raised concern for Maffucci syndrome; however, the absence of enchondromas and histopathologic confirmation of benign cavernous vascular malformations supported KTS. Progressive symptomatic masses were treated with surgical debulking, drainage, wound care, and culture‐directed antibiotics. Recovery was uneventful, with continued conservative surveillance. This case shows how combined clinical assessment, Doppler ultrasonography, magnetic resonance imaging, and histopathology can distinguish atypical pediatric KTS from skeletal dysplasias and guide treatment when genetic testing and multidisciplinary vascular services are limited.

## Key Clinical Message

Deep intramuscular vascular malformations in pediatric Klippel‐Trenaunay syndrome may mimic Maffucci syndrome when calcifications are present. Absence of enchondromas, supported by Doppler ultrasonography, magnetic resonance imaging, and histopathology, can establish the diagnosis and guide surgical planning, especially when molecular testing is unavailable.

## 1 Introduction

Klippel‐Trenaunay syndrome is a complex and rare congenital disorder characterized by capillary, venous, and lymphatic malformations and excessive limb growth [1]. The exact etiology of the disease remains unknown; however, both genetic and environmental factors have been proposed as contributing factors. A somatic mutation in the PIK3CA gene has also been linked to the disease [2]. It presents as a classic triad of capillary malformations (port‐wine stains), venous abnormalities (varicosities), and bone and soft tissue hypertrophy. Patients experience a wide range of symptoms, which include pain, limb swelling, lymphedema, spontaneous bleeding, superficial vein swelling, and increased risk of deep venous thrombosis. The lower extremities are more prone to the disease's vascular manifestations. The diagnosis is usually made with the presence of physical findings, along with supportive evidence of segmental port‐wine stain, lymphatic and venous malformations, and overgrowth on imaging [2]. Maffucci syndrome is another overgrowth syndrome that is similar to KTS and is characterized by venous malformations, capillary hemangiomas, and, in some instances, lymphangiomas; however, these patients over time develop multiple enchondromas. Enchondromas are benign tumors of cartilage and have up to a 17%–30% risk of conversion to chondrosarcomas [3].

## 2 Case History

A 12‐year‐old fully vaccinated boy presented with a longstanding history of right lower limb swelling since birth, complicated by recurrent left calf cellulitis and multiple prior debulking surgeries. His condition has progressively worsened with limb enlargement, recurrent ulcerations, and chronic purulent discharge. To date he has undergone four soft tissue excisions and required several blood transfusions to address anemia secondary to his chronic wound burden. Given the characteristic clinical presentation, a working diagnosis of Klippel–Trenaunay Syndrome (KTS) was established, prompting comprehensive radiologic evaluation to delineate the extent of vascular and soft tissue involvement. The clinical course is summarized chronologically in Table 1.

**TABLE 1:** Timeline of the clinical course.

Date (approx.)	Event	Findings and management
Birth	Right lower‐limb swelling and cutaneous vascular lesions	Present from birth
2022	First presentation to our emergency department; initial work‐up	Swelling, ulceration, chronic purulent discharge. Microcytic anemia (Hb 8.8–9.7 g/dL), ESR 100 mm/h, procalcitonin 1.82 ng/mL; sterile blood and urine cultures. Doppler: calcified cavernous lesions, superficial venous reflux, incompetent perforators, patent deep veins, triphasic arterial flow. Radiographs: soft‐tissue hypertrophy, no enchondromas. MRI: gastrocnemius and tibialis involvement. Abdominal ultrasound: no visceral malformation
2022–2024	Recurrent admissions	Four soft‐tissue excisions; several blood transfusions for anemia related to chronic wounds
2024	Repeat Doppler and radiographs	Triphasic arterial waveforms bilaterally, no deep vein thrombosis, mild to moderate right‐sided arterial insufficiency; limb‐length discrepancy
2024	Surgical debulking	10 × 10 cm and 6 × 4 cm masses excised; 2 drains; primary closure. Histopathology: Benign cavernous vascular malformation
Postoperative admission	Wound infection treated	Culture: *Pseudomonas aeruginosa* . Culture‐directed antibiotics, twice‐daily dressings; uneventful recovery
After discharge	Outpatient review	Conservative management; structured long‐term follow‐up data not available

On clinical examination the child appeared irritable, likely due to discomfort from pain. On inspection of the lower body, there were multiple nodular swellings with clear ulcerated lesions affecting both legs, most noticeably on the back of the right calf and foot, with similar involvement on the left foot and leg. The lesions showed no warmth or tenderness. The child also had distinctive skin lesions on the right side and buttocks, consistent with vascular malformations. These symptoms have been present since birth. The condition first came to medical attention at our center in 2022, when the child was brought to the emergency department with these complaints. Since then, he has had multiple hospital admissions for the same issues. The right lower limb was visibly bulkier than the left, with macrodactyly of the right foot and a clinically apparent limb‐length discrepancy that was not formally measured. On the anteroposterior radiograph, the maximal transverse soft‐tissue diameter of the right calf was approximately 12 cm, against approximately 9 cm on the left (estimated using tibial length as the reference scale) (Figure 1). The child had also developed progressive weakness of the right leg as the swelling enlarged; muscle power was not formally graded. No other organ system was involved.

**FIGURE 1:**
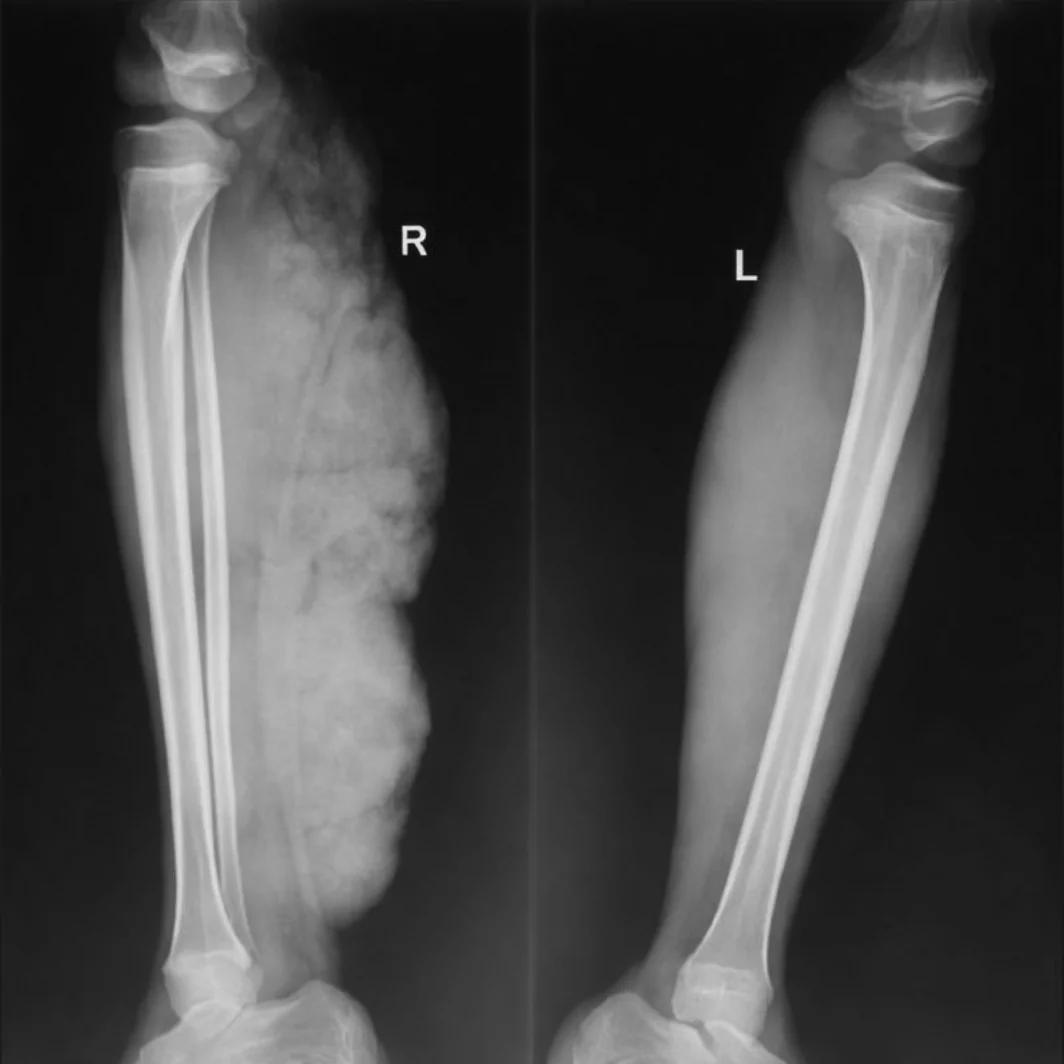
Anteroposterior (AP) radiograph of bilateral lower legs in a 12‐year‐old child with clinically diagnosed Klippel–Trénaunay Syndrome. The right lower limb (R) shows marked medial soft‐tissue hypertrophy and swelling, while the left limb (L) demonstrates preserved soft‐tissue planes and normal tibia–fibula anatomy. No osseous lesions, enchondromas, or cortical destruction are visualized, helping exclude differentials such as Maffucci Syndrome. The deep compartments show no radiographic evidence of bone involvement.

Serial laboratory evaluations showed a persistent microcytic anemia (Hb 8.8–9.7 g/dL; MCV ~76–79 fl) with elevated RDW (46 fL), intermittent leukocytosis (TLC up to 23.4 × 10^3/uL, neutrophil predominant), and occasional mild thrombocytosis (410 × 10^3/uL). Inflammatory markers were raised with ESR at 100 mm/h and procalcitonin mildly elevated at 1.82 ng/mL. Renal and liver function tests, along with electrolytes, remained within normal limits. Urinalysis showed red‐colored urine; however, both urine and blood cultures were sterile. Overall, these findings pointed to a chronic inflammatory state with anemia of chronic disease, in the absence of systemic infection or organ dysfunction.

Initial color Doppler ultrasound of the right lower limb demonstrated marked soft tissue hypertrophy with macrodactyly. Multiple hypoechoic lesions containing cystic spaces and calcifications were noted along the medial and posterior thigh, extending into the posterior calf and ankle, with the largest measuring 5.5 × 2.5 cm. Color Doppler confirmed venous flow within these lesions, consistent with cavernous hemangiomas. Extensive superficial venous dilatations and incompetent perforators were observed throughout the calf and ankle, accompanied by reversal of flow at the common femoral vein and saphenofemoral junction on Valsalva maneuver. Importantly, the deep veins remained patent without thrombosis, and arterial flow was preserved with triphasic waveforms.

Plain radiographs confirmed right lower‐limb soft‐tissue hypertrophy without underlying osseous involvement, enchondromas, or other destructive bony lesions (Figure 1). An anteroposterior abdominopelvic radiograph showed no osseous lesions, enchondromas, or abnormal calcifications in the spine or pelvis (Figure 2). Together, these findings supported exclusion of Maffucci syndrome.

**FIGURE 2:**
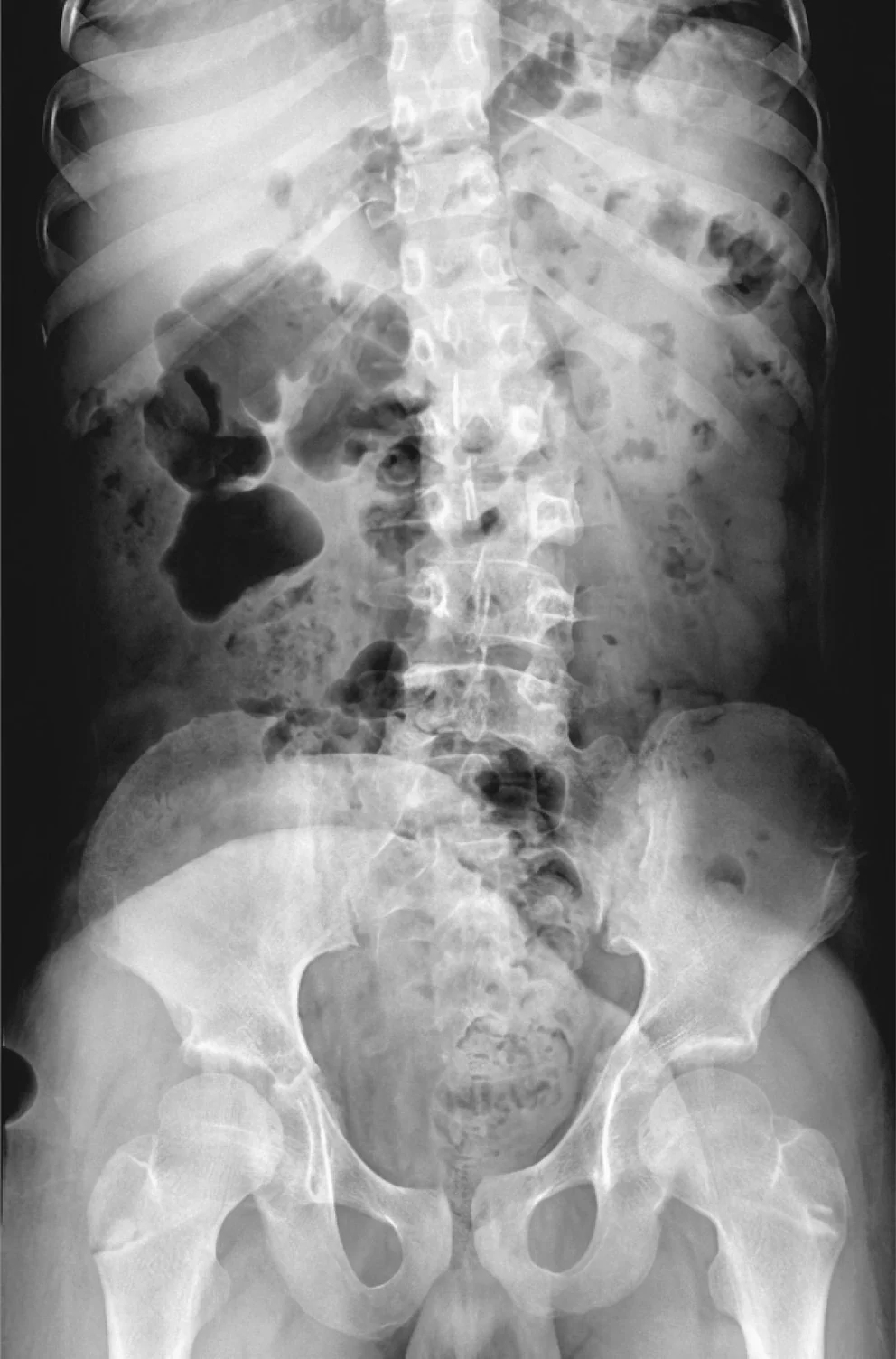
Anteroposterior (AP) abdominal–pelvic radiograph demonstrating normal alignment of the thoracolumbar spine and intact pelvic girdle. Bowel gas pattern is non‐obstructive, with moderate fecal loading in the rectosigmoid colon. No osseous lesions, abnormal calcifications, or structural deformities are visualized.

Further characterization with contrast‐enhanced MRI of the right leg revealed diffuse intramuscular involvement of the proximal gastrocnemius and tibialis muscles with loss of normal architecture. Extensive subcutaneous edema and multiple large cystic spaces appeared hyperintense on T2‐weighted images, in keeping with complex vascular malformations. There was no evidence of marrow infiltration or joint involvement. To exclude visceral extension, an abdominal ultrasound demonstrated normal hepatic and splenic sizes, patent portal circulation, and only mild renal parenchymal changes, without visceral vascular malformations.

His symptoms worsened over the following months. A repeat arterial and venous Doppler study in 2024, about 2 years after the first, again showed patent arteries with triphasic waveforms bilaterally and no deep vein thrombosis. The report described mild to moderate arterial insufficiency in the right lower limb, without loss of the triphasic waveform. Bilateral soft tissue edema extended to the dorsum of the feet. Plain radiographs confirmed the limb length discrepancy and extensive soft tissue hypertrophy without underlying osseous involvement.

Histopathological examination of the excised specimens confirmed benign vascular malformations composed of cavernous spaces interspersed within hypertrophied soft tissue, without evidence of malignancy. These findings corroborated the clinical and radiologic diagnosis of Klippel–Trenaunay Syndrome.

Given the progressive enlargement of posterior thigh and ankle swellings and persistent chronic purulent discharge, the child underwent surgical debulking in 2024. Under local anesthesia, two substantial masses were excised—a 10 × 10 cm lesion from below the popliteal fossa and a 6 × 4 cm mass approximately 2 cm above the ankle. Intraoperatively, two drains were placed to reduce the risk of postoperative seroma formation, and the wounds were closed primarily. Inpatient treatment includes injection ceftriaxone 2.5 g IV once daily, injection vancomycin 350 mg IV, injection Mgso4 1500 mg IV, and injection Tanzo 3500 mg IV 8 hourly.

Postoperative care included meticulous wound management and tailored antibiotic therapy guided by culture, which grew *Pseudomonas aeruginosa* sensitive to cefepime, imipenem, and piperacillin‐tazobactam. He was prescribed a regimen including Augmentin DS syrup (250 mg/5 mL) twice daily and Brufen DS syrup (15 mL) thrice daily for pain management. Twice daily dressing with KMNO_4_‐soaked gauzes and fusidic cream was advised to prevent infection and promote healing. The immediate postoperative course was uneventful.

### 2.1 Follow‐Up and Outcome

Both drains were removed and the wounds were dressed twice daily until discharge. The *Pseudomonas aeruginosa* wound infection resolved on culture‐directed antibiotics, and no other postoperative complication, such as seroma, hematoma, wound dehiscence, or deep vein thrombosis, was recorded during the admission. After discharge, he continued on conservative management with outpatient review to monitor for recurrence and complications. Structured long‐term data on wound healing, recurrence, and limb function were not available at the time of writing, and we acknowledge this as a limitation.

## 3 Discussion

Klippel‐Trénaunay Syndrome (KTS) is a rare congenital vascular disorder with an estimated prevalence ranging from 2:100,000 to 5:100,000 live births, characterized by the classical triad of capillary malformations, venous/lymphatic anomalies, and tissue/limb overgrowth [1]. Although KTS typically manifests with superficial involvement, with port‐wine stains occurring in 98% of cases and varicose veins present in 72% of patients, rare extensions into deep intramuscular structures and arterial circulation have been rarely reported. According to a study, deep intramuscular vascular malformations are quite rare, believed to be less than 1% of all vascular malformations [4]. Our patient presented with extensive intramuscular vascular malformations involving the gastrocnemius and tibialis muscles. Literature indicates that such deep venous system anomalies in KTS include duplication, aplasia, hypoplasia, and aneurysmal dilatation of affected veins, with the most common deep venous abnormalities affecting the popliteal and femoral veins. When these deep intramuscular extensions occur, isolated reports have documented associated complications such as pain, progressive weakness, and impaired growth, likely due to venous hypertension and local tissue hypoxia from vascular steal phenomena [5]. The gastrocnemius muscle's biarticular anatomy and higher proportion of Type II fibers may predispose it to such complications in KTS. Type II fibers, being more susceptible to eccentric‐induced injury, may explain the patient's progressive weakness and abnormal growth patterns [6]. To date, there is minimal literature detailing progressive intramuscular involvement of both the gastrocnemius and tibialis muscles in KTS, despite the unique functional and structural vulnerabilities of these compartments. Our case adds a documented example of combined gastrocnemius and tibialis involvement in a child, with progressive weakness and asymmetric limb growth documented in the case description.

A notable diagnostic dilemma in this case was the initial misclassification as Maffucci Syndrome, a rare skeletal dysplasia characterized by enchondromas with vascular malformations [3]. No formal consensus criteria exist for Maffucci syndrome. The diagnosis is clinical and radiologic and needs two components: multiple enchondromas (enchondromatosis), usually asymmetric and involving the short tubular bones of the hands and feet and the metaphyses of long bones, together with soft‐tissue vascular lesions, typically spindle cell hemangiomas or venous malformations that often contain phleboliths [7, 8]. Somatic mosaic IDH1 or IDH2 mutations are found in the lesions of about 77% of patients and support the diagnosis when tissue is tested [8]. Enchondromatosis without vascular lesions is Ollier disease; vascular lesions without enchondromas do not meet the definition of Maffucci syndrome. The presence of cavernous hemangiomas with calcifications in the thigh, calf, and ankle closely mimicked Maffucci lesions on imaging. These lesions satisfied only the vascular component. Radiographs showed no enchondromas or other bone or cartilage lesion, so the skeletal criterion was not met and the differential was excluded. This real‐world misinterpretation highlights the overlap of vascular dysplasias on imaging and reinforces the role of clinicoradiologic correlation and histopathology in differentiating these entities. Importantly, distinguishing between these syndromes carries prognostic weight: Maffucci Syndrome carries a 17%–30% risk of malignant transformation to chondrosarcoma [3], and a multicenter series of 161 patients with enchondromatosis reported chondrosarcoma in 40% [9], whereas KTS rarely progresses to malignancy [3].

Combined Doppler ultrasound and MRI defined the disease in this patient. Doppler showed venous reflux at the saphenofemoral junction and incompetent perforating veins, findings usually discussed in adult chronic venous insufficiency and less often described in this detail in children. MRI showed the intramuscular extent of the malformations, with hyperintense cystic spaces on T2‐weighted images, and this guided surgical planning [10]. This pairing is standard practice in vascular anomaly centers. In a setting without interventional radiology or genetic testing, it was the main tool available to map the lesions before surgery.

Chronic venous insufficiency and valvular incompetence are well recognized in adults with KTS and receive less attention in children. Our patient had reflux at the common femoral vein and saphenofemoral junction on Valsalva, with incompetent perforators in the calf and ankle. Arterial waveforms stayed triphasic on both the initial and the follow‐up Doppler, which argues against significant arteriovenous shunting or proximal arterial occlusion. The mild to moderate right‐sided arterial insufficiency on the follow‐up study should be read with that in mind; it may reflect reduced distal perfusion within hypertrophied, malformed muscle and soft tissue rather than a fixed arterial lesion. Venous hypertension and local tissue hypoxia have been proposed as causes of pain and disturbed growth in limbs with extensive vascular malformations [5], and deep vein thrombosis is reported in about 8% of patients with KTS [11]. Children with deep intramuscular KTS therefore need repeated Doppler surveillance, even when the deep veins are patent at baseline.

This case also reflects broader systemic inequities faced by children with rare vascular disorders, particularly within resource‐limited healthcare environments. Our patient underwent multiple hospitalizations and debulking surgeries before a definitive diagnosis was established, an outcome not merely of clinical complexity but of structural barriers to comprehensive care. Limited access to advanced imaging modalities, constrained availability of multidisciplinary expertise, and the absence of molecular diagnostic tools such as PIK3CA mutation testing all contributed to diagnostic delay and fragmented management. Globally, patients with rare diseases experience an average diagnostic delay of 5–8 years, a pattern echoed in our setting, where specialized genetic testing remains inaccessible [12]. These inequities exacerbate disease progression, increase procedural morbidity, and impose a significant psychosocial and economic burden on affected families. Bridging this gap requires the integration of multidisciplinary vascular anomaly teams, expansion of molecular diagnostic capacity, and inclusion of rare diseases in national health policy frameworks to promote timely, equitable care.

## 4 Limitations

This report has limitations. PIK3CA mutation analysis, the molecular basis of KTS within the PIK3CA‐related overgrowth spectrum (PROS) [13], was not performed. The test is not available at our center. KTS‐associated PIK3CA variants are somatic and often present at low allele fractions, so they are best detected by deep sequencing of lesional tissue rather than blood, which would have meant sending tissue to a laboratory abroad; this was not feasible in this case. IDH1/IDH2 testing, which would have given molecular support for excluding Maffucci syndrome, was unavailable for the same reason. The diagnosis therefore rests on clinical, radiologic, and histopathologic findings. Structured follow‐up data after discharge were not available, so long‐term recurrence, wound outcome, limb function, thromboembolic events, and chronic venous hypertension could not be assessed. Limb‐length discrepancy and muscle power were not formally measured, and soft‐tissue dimensions were estimated from radiographs. Comparison with other cases is limited because pediatric intramuscular KTS is rare and published data come mostly from isolated case reports and small series. Finally, this is a single patient from one tertiary‐care center in a resource‐limited setting, and the findings may not apply elsewhere.

## 5 Conclusion

This case delineates an exceptionally complex pediatric presentation of Klippel‐Trénaunay Syndrome characterized by deep intramuscular vascular malformations, severe venous insufficiency with valvular incompetence, and mild to moderate arterial insufficiency with preserved triphasic waveforms, features rarely documented in this age group. The initial diagnostic overlap with Maffucci Syndrome underscores the need for careful clinicoradiologic correlation and histopathologic confirmation in ambiguous vascular overgrowth syndromes. Multimodal imaging proved indispensable for delineating disease extent and guiding surgical strategy, and remains the practical standard where molecular testing is unavailable. The case also reflects persistent inequities in rare‐disease diagnosis and management in low‐resource settings, emphasizing the urgent need for equitable access to multidisciplinary expertise and genetic testing. Early recognition and precise differentiation of atypical KTS variants are critical to reducing morbidity, preventing unnecessary interventions, and paving the way for precision‐based therapies such as PIK3CA‐targeted agents in the future.

## Author Contributions

**Syeda Simrah Shah:** conceptualization, methodology, project administration, visualization, writing – original draft. **Barka Sajid:** investigation, validation, writing – review and editing, writing – original draft, supervision. **Muzainah Tabassum:** writing – original draft, writing – review and editing, resources. **Mirha Ali:** writing – review and editing, writing – original draft, visualization. **Taher Janoowala:** writing – review and editing, writing – original draft. **Tehreem Farooq:** writing – review and editing, writing – original draft, methodology, validation. **Muhammad Ahsan:** writing – review and editing, writing – original draft, project administration. **Mirza Mohammad Ali Baig:** writing – original draft, writing – review and editing. **Mirza Daniyal Baig:** writing – original draft, writing – review and editing. **Mohammed Hammad Jaber Amin:** writing – review and editing.

## Funding

The authors have nothing to report.

## Ethics Statement

This case report was conducted in accordance with the ethical standards of the institutional review board. Formal approval was waived for this retrospective case study as per institutional guidelines.

## Consent

Written informed consent was obtained from the patient and legal guardian for the publication of this case report, including de‐identified clinical details and images.

## Conflicts of Interest

The authors declare no conflicts of interest.

## Data Availability

Data sharing not applicable to this article as no datasets were generated or analyzed during the current study.
